# Value Congruence Regarding Patient Safety Among Nurses and Their Managers and Its Association With Patient Safety Behaviors: The Moderating Role of Psychological Safety

**DOI:** 10.7759/cureus.97891

**Published:** 2025-11-26

**Authors:** Ayano Fujiyoshi-Ito, Yasuko Ogata, Yoshie Yumoto, Miki Sasaki, Yuki Yonekura

**Affiliations:** 1 Department of Nursing Management and Gerontology Nursing, Graduate School of Health Care Sciences, Institute of Science Tokyo, Tokyo, JPN; 2 Department of Nursing Informatics, Graduate School of Nursing Science, St. Luke's International University, Tokyo, JPN

**Keywords:** leadership, nurses, organizational psychology, patient safety, patient safety culture, patient safety improvement, psychological safety, quality improvement, quality patient care, safety behaviors

## Abstract

Introduction

Securing patient safety is a global concern. Unsafe care can cause preventable adverse events. Nurses play a key role through two types of patient safety behaviors: compliance (mandatory adherence to safety protocols) and participation (voluntary efforts to improve safety). While value congruence with supervisors promotes positive work behaviors, its influence on patient safety behaviors remains unclear. Moreover, a clear link between psychological safety and patient safety outcomes is yet to be established. To fill this gap, the present study examines whether psychological safety moderates the relationships between value congruence regarding patient safety and patient safety behaviors.

Objectives

To examine the associations between nurses’ value congruence with their managers regarding patient safety and both patient safety compliance and participation, and to assess the moderating role of psychological safety in these relationships.

Design

A cross-sectional, multicenter, self-administered questionnaire survey.

Materials and methods

The survey was conducted from April to May 2025 among registered nurses working in hospitals across Japan with more than 200 beds. Multivariate regression analyses were employed to examine the relationships among patient safety behaviors, value congruence, and psychological safety. This study was conducted in accordance with the STROBE (STrengthening the Reporting of OBservational Studies in Epidemiology) guidelines.

Results

In total, 509 nurses at six hospitals were included in the study. Value congruence was associated with patient safety compliance (β=0.347, p<0.001) and participation (β=0.341, p<0.001). The interaction effect of value congruence and psychological safety on patient safety compliance and participation yielded β=0.020 (p<0.630) and β=0.081 (p=0.047), respectively.

Conclusions

This study introduced the novel concept of value congruence as a contributor to patient safety. Our findings suggest that sharing values and goals related to patient safety can promote both types of patient safety behaviors, and psychological safety plays a key role in fostering voluntary engagement in patient safety practices.

## Introduction

Ensuring patient safety is a global concern. Despite worldwide efforts, improving patient safety remains a persistent challenge. Unsafe care, including medication errors, communication breakdowns, or non-adherence to safety procedures, is a cause of adverse events in patients, such as death, disability, and disease [1]. Every year, approximately 130 million adverse events occur in hospitals in low- and middle-income countries, resulting in about 2.6 million deaths [1]. In contrast, in high-income countries, unsafe care accounts for roughly 15% of health spending to manage the consequences of patient harm [1]. The indirect costs of unsafe care are approximately US$3,400 per patient experiencing at least one case of patient harm [1]. Therefore, unsafe care practices lead to individual harm and pose a serious burden on global health systems.

Nurses play a key role in promoting patient safety and collaborating with other healthcare professionals to reduce the risk of incidents. The quality of nursing care has been shown to be associated with patient outcomes, such as nosocomial infections and mortality [2].

In the safety science literature, safety behavior is considered a performance indicator [3]. Safety behavior, defined as an action to promote safety, consists of two types: safety compliance and safety participation. In the context of patient safety, these are recognized as patient safety compliance and patient safety participation, respectively [4]. Patient safety compliance refers to mandatory activities involving adherence to specific procedures [4,5]. Examples include adherence to procedures for the use of personal protective equipment and hand hygiene. Patient safety participation refers to voluntary behaviors aimed at enhancing patient safety, often involving individuals putting in extra effort [4,5]. Examples include helping others, speaking up about patient safety issues, cautioning others about unsafe behaviors, and sharing knowledge.

To better understand what drives nurses’ patient safety behaviors, it is important to consider the internal and environmental factors that influence nurses’ actions. Lewin’s field theory offers a framework for understanding the mechanisms underlying human behavior [6], whereby behavior is determined by the interaction between an individual’s internal factors and environmental factors, rather than by the independent effects of each factor [6]. The individual’s internal factors include perceptions, values, goals, attitudes, and experiences. Environmental factors refer to the entire context surrounding a person. In organizational research, these include an organization, a group, and supervisors [7]. Nurses’ behaviors may also be explained by field theory. Previous research has demonstrated that internal and environmental factors are linked to patient safety behavior. Studies have reported that individual values and motivation related to patient safety (individual internal factors) were associated with patient safety behavior [4,8]. Furthermore, nurse managers (supervisors) were found to influence the patient safety behaviors of their subordinates (nurses) [9]. Also, nurse managers’ values and attitudes toward patient safety (environmental factors) were associated with nurses’ patient safety behaviors [10]. Therefore, focusing on individual values toward patient safety and the role of the supervisor (the interaction between an individual’s internal and environmental factors) may be important to understand the patient safety behaviors of subordinates. This study conceptualized the value of patient safety as an individual internal factor and the dyadic relationship with their supervisor as an environmental factor and examined the impact of this relationship on nurses’ patient safety behaviors.

Previous studies have focused on either individual internal factors or environmental factors. However, the relationship between internal and environmental factors and safety behavior remains insufficiently explored. To address this gap, this study examined value congruence between nurses and their supervisors (nurse managers) regarding patient safety and its impact on nurses’ patient safety behaviors.

The congruence in values regarding patient safety between nurses and their supervisors can be understood in terms of person-supervisor fit. Person-supervisor fit refers to the degree of subjective perception of agreement between an individual’s values, goals, personality traits, or attitudes and those of their supervisor, and is a component of the multidimensional person-environment fit [7]. Previous studies suggested that when perceived fit is high, employees recognize shared goals and values with their supervisor, which enhances their motivation and engagement, leading to desirable behaviors that align with those goals and values [11]. While the influence of value congruence on patient safety behaviors remains unclear, previous research revealed a potential link between value congruence among nurses and managers and patient safety through interviews with nurses [12]. Therefore, this study addresses this gap by empirically examining the association between value congruence and nurses’ patient safety behaviors. We hypothesized that nurses are more likely to be motivated to engage in patient safety behaviors consistent with their values when their values and attitudes toward patient safety align with those of their nurse managers: H1-a: Value congruence regarding patient safety between nurses and their nurse managers is positively associated with nurses’ patient safety compliance; H1-b: Value congruence regarding patient safety between nurses and their nurse managers is positively associated with nurses’ patient safety participation.

However, even when congruence is high, nurses do not always engage in patient safety behaviors because they may fear the consequences of their actions, and a punitive culture contributes to these concerns [13]. In other words, nurses might not commit to patient safety behaviors when they fear negative consequences or work within a punitive culture, even if value congruence with their supervisors exists. Psychological safety plays a role in reducing these barriers. Psychological safety is described as a belief that a team is safe for taking interpersonal risks, even if one might be seen as ignorant, incompetent, negative, or disruptive [14], or as an individual’s perception of the consequences of taking interpersonal risks and of their work environment as characterized by a non-punitive culture and strong interpersonal relationships [15]. Previous studies found that psychological safety promotes specific patient safety behaviors [16]. Furthermore, psychological safety moderated the relationship between others’ values regarding patient safety and nurses’ unsafe practices [17]. Thus, psychological safety could strengthen the interaction effect between person-supervisor fit in terms of patient safety and patient safety behaviors. However, a recent systematic review revealed that the impact of psychological safety on patient safety outcomes remains unclear [18]. To address this research gap, this study examines whether psychological safety moderates the relationships between value congruence regarding patient safety and patient safety behaviors: H2-a: Psychological safety moderates the relationship between value congruence regarding patient safety and nurses’ patient safety compliance; H2-b: Psychological safety moderates the relationship between value congruence regarding patient safety and nurses’ patient safety participation.

## Materials and methods

**Figure 1:**
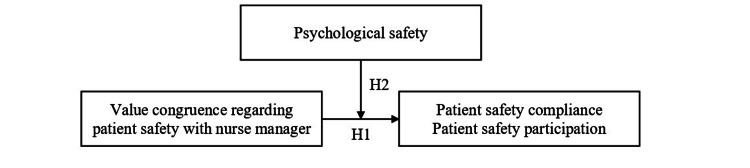
Conceptual model underlying the study H1-a: Value congruence regarding patient safety between nurses and their nurse managers is positively associated with nurses’ patient safety compliance. H1-b: Value congruence regarding patient safety between nurses and their nurse managers is positively associated with nurses’ patient safety participation. H2-a: Psychological safety moderates the relationship between value congruence regarding patient safety and nurses’ patient safety compliance. H2-b: Psychological safety moderates the relationship between value congruence regarding patient safety and nurses’ patient safety participation. H1, Hypotheses 1; H2, Hypotheses 2

This study examined the associations between value congruence regarding patient safety between nurses and their managers and two types of patient safety behaviors, compliance and participation, and investigated the moderating role of psychological safety in these relationships. Figure 1 shows the conceptual model underlying the study.

Design, setting, and participants

A multicenter, cross-sectional survey was administered to nurses working in hospitals across Japan. The inclusion criteria were as follows: hospitals with over 200 beds and registered nurses who provided direct patient care, had at least one year of nursing experience, and were not in a managerial position. Newly qualified nurses with less than one year of nursing experience were excluded. Eligible hospitals were stratified by the number of beds, and 50 hospitals were randomly sampled based on bed size. Of the selected hospitals, six agreed to participate in the survey.

Anonymous, paper-based, self-administered questionnaires were distributed to nurses (n=1,734) at the participating hospitals. The survey was conducted from April 17 to May 7, 2025.

Measurements

Patient Safety Compliance and Patient Safety Participation

Two types of behaviors were measured based on the items of Neal’s Safety Behavior [5]. The items were translated into Japanese and modified to assess safety behaviors in the context of patient safety. Two professional Japanese-English translators, each with more than 10 years of experience specializing in healthcare management and nursing, independently conducted the translation process. One performed the forward translation, and the other conducted the back translation. The equivalence of the translations was reviewed. Subsequently, a cognitive debriefing on the translated items in the context of patient safety was conducted. This procedure allowed the authors to evaluate the appropriateness of alternative wordings, the comprehensibility of items, the interpretation by participants, and the overall validity for the target population. The final Japanese version was finalized through consensus among the authors (see Figure 3 in Appendices). Confirmatory factor analysis was conducted to examine whether the scale demonstrated the same factor structure as the original version. The results supported the factorial validity of the scale (goodness-of-fit index=0.952; adjusted goodness-of-fit index=0.910; comparative fit index=0.960; standardized root mean square residual=0.723).

Each behavior comprises four items rated on a scale from 1 (=strongly disagree) to 5 (=strongly agree). Scores were calculated by dividing the total score for each behavior by the number of items. Cronbach’s alpha for patient safety compliance and patient safety participation was 0.883 and 0.826, respectively.

Value Congruence Toward Patient Safety Between Nurses and Their Managers

Value congruence regarding patient safety between nurses and their managers was measured using a modified version of the person-supervisor fit subscale (three items) of the validated Japanese version of the Perceived Fit Scale [19,20], which consists of five dimensions: needs-supplies, demands-abilities, person-organization, person-group, and person-supervisor fits. While the original person-supervisor fit subscale was designed to assess value congruence with the supervisor in a general life context, the items were adapted to specifically address value congruence with nurse managers in the context of patient safety (see Figure 3 in Appendices). Items were rated on a scale from 1 (=completely disagree) to 7 (=completely agree). The overall score was calculated as the mean of all individual item scores. Cronbach’s alpha was 0.957.

Psychological Safety

Psychological safety was measured using the validated Japanese version of the psychological safety scale for workers (see Figure 3 in Appendices) [21,22]. This scale assesses employees’ perceptions of interpersonal risk-taking in the workplace. The scale comprises five items, rated on a scale from 1 (=strongly disagree) to 5 (=strongly agree). The overall score for psychological safety was calculated as the mean of all individual item scores. Cronbach’s alpha was 0.819.

Demographic Characteristics

The survey collected data on demographic characteristics, including age, gender, educational background, involvement in patient safety roles (0=no, 1=yes), unit type, years of nursing experience, and years in the current unit. The participating hospitals were categorized by the number of beds (≤399 beds vs. ≥400). In Japan, hospitals with 400 or more beds include designated advanced-function hospitals (special-function hospitals) required to provide advanced care and maintain an advanced patient safety management system. Therefore, hospitals were divided into those with fewer than 400 beds and those with 400 or more beds to distinguish between hospitals with different organizational characteristics relevant to patient safety.

Data analysis

Descriptive analyses were conducted to summarize the variables. Multivariate regression analyses were performed to examine the relationships among value congruence regarding patient safety, psychological safety, and the two types of patient safety behaviors (patient safety compliance and participation). Four models were constructed for each patient safety behavior. Model 1 included the main effects of value congruence regarding patient safety. Model 2 included the main effects of both value congruence and psychological safety. Model 3 added the interaction effect of psychological safety on the relationship between value congruence and the two types of patient safety behaviors. To visualize the interaction effect of value congruence and psychological safety on each patient safety behavior in Model 3, a two-way analysis of variance was conducted for group comparison and to assess the main and interaction effects on patient safety behaviors. Value congruence and psychological safety were categorized into two groups based on ±0.5 SD from the mean (low: mean ≤-0.5 SD; high: mean ≥0.5 SD). All models were controlled for the number of hospital beds, years of nursing experience, and involvement in patient safety roles, factors previously identified as associated with patient safety behaviors. Before conducting the analyses, listwise deletion was employed to exclude participants with missing data on variables used in the analysis, and all continuous variables were mean-centered. Statistical analyses were conducted using SPSS for Windows (version 29.0) (IBM Corp., Armonk, NY, USA), and statistical significance was set at p<0.05. This study was conducted in accordance with the STrengthening the Reporting of OBservational Studies in Epidemiology (STROBE) guidelines (see Table 4 of Appendices) [23].

Ethical approval and informed consent

This study was approved by the Education Ethics Committee, Institute of Science Tokyo, Japan (No. C2024-014). Participation by hospitals and nurses was voluntary. Informed consent was obtained from nurses before commencing the survey.

## Results

Descriptive statistics

A total of 665 nurses from six hospitals participated in the survey. Of these, valid responses were obtained from 509 nurses after excluding those who did not meet the inclusion criteria: nurses who did not consent to participate (n=37), those in managerial positions (n=36), those with less than one year of nursing experience (n=11), those without a registered nurse qualification (n=8), those not involved in direct patient care (n=27), and those with missing data (n=37).

Of the six hospitals, two had ≥400 beds and four had ≤399 beds. Table 1 shows the demographic characteristics and scores of the key variables. Most nurses were female, held associate or diploma degrees, and worked in general inpatient or outpatient departments.

**Table 1:** Summary of demographic characteristics and core variables (n=509)

	n (%)	Mean (SD)
Age (years)		
≤29	170 (33.4)	
30-39	122 (24.0)	
40-49	129 (25.3)	
≥50	75 (14.7)	
Missing	13 (2.6)	
Gender		
Female	473 (92.9)	
Male	34 (6.7)	
Other/missing	2 (0.4)	
Educational background		
Associate or diploma degree	394 (77.4)	
Bachelor’s degree	115 (22.6)	
Involvement in patient safety roles		
Yes	111 (21.8)	
No	398 (78.2)	
Unit type		
Emergency, critical care, or operating room	77 (15.1)	
General inpatient/outpatient	391 (76.8)	
Missing	41 (8.1)	
Nursing experience (years)		14.14 (9.60)
Patient safety compliance		4.04 (0.62)
Patient safety participation		3.38 (0.70)
Value congruence in patient safety		4.69 (1.05)
Psychological safety		3.40 (0.74)

Hypothesis testing

According to Table 2, value congruence toward patient safety between nurses and their managers had positive and significant relationships with patient safety compliance (β=0.347, p<0.001), and value congruence remained positively associated with patient safety compliance when controlling for psychological safety (β=0.302, p<0.001). However, the interaction between value congruence and psychological safety was not significant (β=0.020, p=0.630).

**Table 2:** Associations between patient safety compliance, value congruence, and psychological safety (n=509)

Dependent variable: patient safety compliance	Model 1			Model 2			Model 3		
	β	p-value	95% CI	β	p-value	95% CI	β	p-value	95% CI
Value congruence	0.347	<0.001	0.265 to 0.428	0.302	<0.001	0.213 to 0.390	0.301	<0.001	0.212 to 0.390
Psychological safety				0.111	0.014	0.022 to 0.201	0.110	0.016	0.021 to 0.199
Value congruence × psychological safety							0.020	0.630	-0.061 to 0.101
Number of beds	0.140	0.001	0.059 to 0.221	0.129	0.002	0.048 to 0.210	0.128	0.002	0.047 to 0.209
Nursing experience (years)	0.059	0.158	-0.023 to 0.140	0.069	0.097	-0.012 to 0.150	0.067	0.105	-0.014 to 0.149
Involvement in patient safety roles	0.039	0.347	-0.042 to 0.121	0.039	0.350	-0.042 to 0.120	0.039	0.346	-0.042 to 0.120

**Figure 2:**
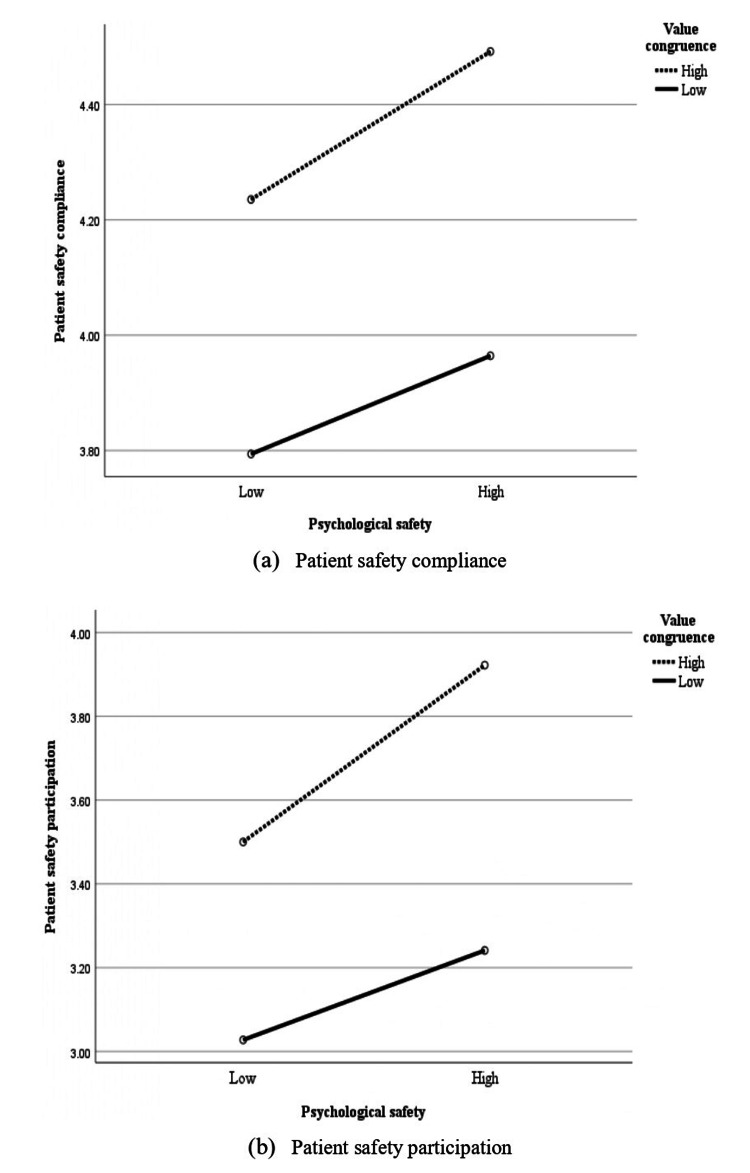
Interactional effect of value congruence and psychological safety on (a) patient safety compliance and (b) participation

**Figure 3:**
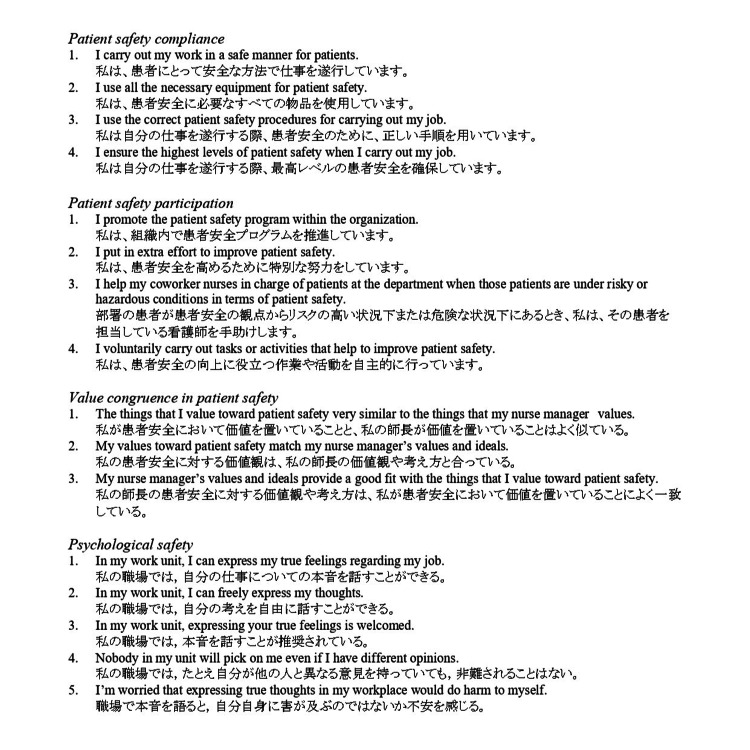
Survey measurements

Table 3 shows that value congruence was positively and significantly associated with patient safety participation (β=0.341, p<0.001), and value congruence remained positively associated with patient safety participation when controlling for psychological safety (β=0.276, p<0.001). Additionally, the interaction between value congruence and psychological safety was significant (β=0.081, p=0.047). Figure 2 illustrates the interaction effect of value congruence and psychological safety on patient safety compliance and participation.

**Table 3:** The interrelationship between patient safety participation, value congruence, and psychological safety (n=509)

Dependent variable: patient safety participation	Model 1			Model 2			Model 3		
	β	p-value	95% CI	β	p-value	95% CI	β	p-value	95% CI
Value congruence	0.341	<0.001	0.260 to 0.422	0.276	<0.001	0.189 to 0.364	0.274	<0.001	0.187 to 0.362
Psychological safety				0.160	<0.001	0.072 to 0.248	0.155	0.001	0.067 to 0.243
Value congruence × psychological safety							0.081	0.047	0.001 to 0.161
Number of beds	0.076	0.065	-0.005 to 0.156	0.060	0.139	-0.020 to 0.140	0.055	0.178	-0.025 to 0.135
Nursing experience (years)	0.110	0.008	0.029 to 0.190	0.124	0.002	0.044 to 0.205	0.119	0.004	0.039 to 0.199
Involvement in patient safety roles	0.121	0.004	0.040 to 0.202	0.120	0.003	0.040 to 0.200	0.121	0.003	0.041 to 0.201

## Discussion

Hypothesis testing

This study examined the associations between nurses’ value congruence with their managers regarding patient safety and two types of patient safety behaviors, patient safety compliance and participation, and the moderating role of psychological safety in these relationships. Value congruence was positively associated with both patient safety compliance and participation. Psychological safety moderated the positive relationship between value congruence and patient safety participation, but not compliance. These findings supported Hypothesis 1 and provided partial support for Hypothesis 2.

In line with field theory, nurses’ value congruence with their managers regarding patient safety, which represents the interaction between an individual’s internal factors (patient safety value) and environmental factors (nurse manager), was related to both types of patient safety behaviors. Our findings are consistent with the theoretical framework based on field theory [6], which describes that the interaction between an individual's internal factors and environmental factors determines behaviors. Our results were consistent with a qualitative study exploring the link between value congruence and patient safety in the context of nursing [12]. Signaling theory is used here to help interpret these findings [24]. According to signaling theory, the supervisor acts as a strong signaler, communicating the desirable value or expected behavior to the subordinate [25]. These signals increase the motivation and commitment of subordinates and encourage them to take actions that are consistent with their values. Therefore, in this study, nurses received the message (signal) that “the shared value related to patient safety was important” and engaged in patient safety compliance and participation when they perceived a fit in values with their manager regarding patient safety.

Interestingly, the interaction of value congruence and psychological safety was only associated with patient safety participation, suggesting that Hypothesis 2-b was supported, whereas Hypothesis 2-a was rejected. Patient safety compliance is an individual action involving fewer interpersonal risks compared to patient safety participation, which requires collaboration with others. Thus, patient safety compliance behavior was considered to be less influenced by psychological safety, which is related to interpersonal relationships. Notably, patient safety compliance may primarily depend on individual-level determinants. Previous studies found that patient safety compliance was associated with experience in patient safety, knowledge, motivation, and goal orientation [4,26,27].

Conversely, engaging in patient safety participation entails collaborative behaviors that may involve asking questions or raising safety concerns, thereby exposing individuals to interpersonal risks, such as being seen as ignorant, incompetent, disruptive, or negative. Consistent with previous studies showing that psychological safety is associated with certain aspects of patient safety participation [16], this study found that psychological safety influenced patient safety participation. The significant interaction of value congruence and psychological safety with patient safety participation suggests that fostering psychological safety has the potential to enhance patient safety participation, even when nurses perceive low value congruence with their managers.

Implications

These findings offer theoretical contributions and generate novel insights that can support efforts to ensure patient safety. This study is the first to examine the relationships between person-supervisor fit and patient safety compliance and participation, demonstrating the applicability of field theory to patient safety behavior and newly identifying value congruence as a potential means to ensure patient safety. Furthermore, regarding Hypothesis 2, our results indicated that patient safety compliance and participation are driven by different mechanisms. Therefore, distinguishing between these two types of behaviors is essential for a more comprehensive understanding of patient safety behaviors and to identify new strategies to enhance patient safety. This contrasts with the approach in previous studies, which treated patient safety behavior as a single construct, overlooking the individual components [8].

Our study has important clinical implications in ensuring patient safety. Sharing values and goals regarding patient safety among managers and nurses is associated with a signal that “shared values are important for patient safety,” encouraging patient safety compliance and participation among nurses. However, a previous study found that it is important to share specific values and goals that nurses can externalize as part of their daily practice, and simply setting values may not be linked to patient safety behaviors [8]. Additionally, even when the person-supervisor fit regarding patient safety is low, nurse managers can encourage patient safety participation to some extent by increasing psychological safety in the unit. Managers can ensure psychological safety through effective leadership, such as demonstrating consistency between their words and actions [17]. Beyond individual efforts by managers, previous studies have shown that system-level approaches and educational interventions for nurses are necessary to ensure psychological safety. These include incorporating communication training into curricula to prepare for challenging conversations, such as raising safety concerns or pointing out others’ unsafe behaviors [28].

Conversely, individual-level approaches are effective in promoting patient safety compliance. These approaches include training focused on knowledge and attitudes toward patient safety, and on the effectiveness of adhering to safety procedures and rules [29].

Limitations

Although this is the first study to identify the influence of value congruence on patient safety behaviors, it has several limitations. First, the cross-sectional design of this study precludes the establishment of a causal relationship between value congruence and the two types of patient safety behaviors. To address this limitation, future research should employ a longitudinal design. However, a cross-sectional observational design employed in the survey is useful for knowledge building when there is a paucity of evidence on a given research topic.

Second, we relied on participants’ self-reported frequency of patient safety behaviors rather than using data based on the actual frequency of behavior performance or objective observations. While subjective data may be influenced by social desirability bias and non-response bias, they provide valuable insights into nurses’ patient safety behaviors. Previous studies have highlighted the difficulties in directly observing the frequency of actual behavior; furthermore, objective indicators, such as the number of incident reports, do not reflect real-world practice [29]. As all variables were measured using the same self-reported survey, there is a possibility of common method bias. However, to minimize this bias, our survey ensured anonymity, and the measures for independent and dependent variables were placed in physically separated sections of the questionnaire.

Third, it remains unclear which specific values are congruent or incongruent between nurses and their managers. Therefore, qualitative research is necessary to explore the content of these shared values.

Finally, the generalizability of the findings to other settings may be limited. The participation rate of hospitals was low. Nevertheless, we employed random stratified sampling in the recruitment process, which helped enhance representativeness and reduce selection bias. Moreover, patient safety participation is likely to be influenced by the specific cultural context. Asian cultures tend to value collectivism, seniority, and harmonious relationships, which affects the specific type of patient safety participation [30]. Therefore, our results should be applied to other cultural contexts with caution. Further studies, involving multi-country comparative designs or cultural moderation analyses, should be conducted to examine the influence of cultural differences.

## Conclusions

This study newly identified that value congruence concerning patient safety among nurses and their managers was associated with two types of patient safety behaviors, namely mandatory actions involving adherence to safety procedures and rules, and voluntary actions aimed at improving patient safety.

Furthermore, psychological safety strengthened the positive relationship between value congruence and patient safety participation. These results suggest that sharing values and goals related to patient safety is important for fostering nurses' safety behaviors, and psychological safety can encourage nurses’ patient safety participation.

## Appendices

**Table 4:** STROBE statement - checklist of items that should be included in reports of cross-sectional studies *Give information separately for exposed and unexposed groups. Note: An Explanation and Elaboration article discusses each checklist item, providing methodological background and published examples of transparent reporting. The STROBE checklist is best used in conjunction with this article (freely available on the websites of PLoS Medicine at http://www.plosmedicine.org/, Annals of Internal Medicine at http://www.annals.org/, and Epidemiology at http://www.epidem.com/). Information on the STROBE Initiative is available at www.strobe-statement.org. STROBE, STrengthening the Reporting of OBservational Studies in Epidemiology

	Recommendation	Page No.
Title and abstract	1. (a) Indicate the study’s design with a commonly used term in the title or the abstract	1
	1. (b) Provide in the abstract an informative and balanced summary of what was done and what was found	1
Introduction		
Background/rationale	2. Explain the scientific background and rationale for the investigation being reported	1-3
Objectives	3. State-specific objectives, including any prespecified hypotheses	2-3
Methods		
Study design	4. Present key elements of the study design early in the paper	3
Setting	5. Describe the setting, locations, and relevant dates, including periods of recruitment, exposure, follow-up, and data collection	3
Participants	6. (a) Give the eligibility criteria, and the sources and methods of selection of participants	3
Variables	7. Clearly define all outcomes, exposures, predictors, potential confounders, and effect modifiers. Give diagnostic criteria, if applicable	3-4
Data sources/measurement	8. For each variable of interest, give sources of data and details of methods of assessment (measurement). Describe the comparability of assessment methods if there is more than one group*	3-4
Bias	9. Describe any efforts to address potential sources of bias	3-4
Study size	10. Explain how the study size was arrived at	4-5
Quantitative variables	11. Explain how quantitative variables were handled in the analyses. If applicable, describe which groupings were chosen and why	4
Statistical methods	12. (a) Describe all statistical methods, including those used to control for confounding	4
	12. (b) Describe any methods used to examine subgroups and interactions	4
	12. (c) Explain how missing data were addressed	4
	12. (d) If applicable, describe analytical methods taking account of sampling strategy	N/A
	12. (e) Describe any sensitivity analyses	N/A
Results		
Participants	13. (a) Report numbers of individuals at each stage of study, e.g., numbers potentially eligible, examined for eligibility, confirmed eligible, included in the study, completing follow-up, and analyzed*	4-5
	13. (b) Give reasons for non-participation at each stage*	4-5
	13. (c) Consider the use of a flow diagram*	No. Since the details were described in our manuscript, the flow diagram was omitted.
Descriptive data	14. (a) Give characteristics of study participants (e.g., demographic, clinical, social) and information on exposures and potential confounders*	4-5
	14. (b) Indicate the number of participants with missing data for each variable of interest*	4-5
Outcome data	15. Report numbers of outcome events or summary measures*	4-5
Main results	16. (a) Give unadjusted estimates and, if applicable, confounder-adjusted estimates and their precision (e.g., 95% confidence interval). Make clear which confounders were adjusted for and why they were included	5-7. Due to space limitations, we were unable to present the unadjusted model.
	16. (b) Report category boundaries when continuous variables were categorized	N/A
	16. (c) If relevant, consider translating estimates of relative risk into absolute risk for a meaningful time period	N/A
Other analyses	17. Report other analyses done, e.g., analyses of subgroups and interactions, and sensitivity analyses	5-7
Discussion		
Key results	18. Summarize key results with reference to study objectives	7-9
Limitations	19. Discuss limitations of the study, taking into account sources of potential bias or imprecision. Discuss both direction and magnitude of any potential bias	8-9
Interpretation	20. Give a cautious overall interpretation of results considering objectives, limitations, multiplicity of analyses, results from similar studies, and other relevant evidence	7-8
Generalizability	21. Discuss the generalisability (external validity) of the study results	9
Other information		
Funding	22. Give the source of funding and the role of the funders for the present study and, if applicable, for the original study on which the present article is based	12
